# An algorithm for efficient constrained mate selection

**DOI:** 10.1186/1297-9686-43-4

**Published:** 2011-01-20

**Authors:** Brian P Kinghorn

**Affiliations:** 1 School of Environmental and Rural Science, Universiy of New England, Armidale, NSW 2350, Australia

## Abstract

**Background:**

Mate selection can be used as a framework to balance key technical, cost and logistical issues while implementing a breeding program at a tactical level. The resulting mating lists accommodate optimal contributions of parents to future generations, in conjunction with other factors such as progeny inbreeding, connection between herds, use of reproductive technologies, management of the genetic distribution of nominated traits, and management of allele/genotype frequencies for nominated QTL/markers.

**Methods:**

This paper describes a mate selection algorithm that is widely used and presents an extension that makes it possible to apply constraints on certain matings, as dictated through a group mating permission matrix.

**Results:**

This full algorithm leads to simpler applications, and to computing speed for the scenario tested, which is several hundred times faster than the previous strategy of penalising solutions that break constraints.

**Conclusions:**

The much higher speed of the method presented here extends the use of mate selection and enables implementation in relatively large programs across breeding units.

## Background

Mate selection is the process of choosing mating pairs or groups i.e. simultaneous selection and mate allocation of animals entering a breeding program [1]. This can be carried out before mating, to make decisions for the active mating group, but it can also be carried out at other stages. Mate selection can cover almost all of the decisions to be made in a selection program, including culling among juveniles, decisions on semen and embryo collection or purchase, migration of breeding stock, active matings and backup matings. It can also be used to set up investment matings, e.g. assortative matings to invest in increased genetic variation, stock migration to invest in the benefits of better connection, progeny testing to invest in future information, and generation of first-cross females to invest in future maternal heterosis [2-4]. Mate selection does not cover decisions on which animals to measure for which traits, including genotyping decisions, but it can cover most other decisions.

Mate selection analysis results in a mating list, which is used to make the decisions described above. The outcome is driven by an objective function that should include the full range of technical, logistical and cost issues that prevail. This list of motivating issues can be very long, with some examples being genetic gain, genetic diversity, progeny inbreeding, use of reproductive technologies, targeting genotype frequencies for key markers, managing trait distributions, keeping within a budget and not breaking logistical constraints or constraints that reflect the attitudes of the breeder. Mate selection analysis leads to the progressive use of scientific principles in a practical manner that accommodates real constraints, along with practitioner experience and attitudes.

This paper relates to the inclusion of logistical constraints in mate selection analysis, such as lack of ability for a natural mating bull to cover more than a given number of cows, or to operate on more than one farm. In particular, this paper handles constraints related to animal grouping, where matings are not permitted between certain groups. This can be due to

• Geographical separation, or quarantine barriers.

• Perceptions of compatibility, for example where the female group "Heifers" should only be mated with the male group "Low birth weight EBV bulls".

• Cases where "virtual matings" are necessary, for example where immature juveniles are selected as part of a multi-stage selection/culling process, and the male group "juveniles" night only be permitted 'mate' with the female group "juveniles".

Practical experience with mate selection implementations shows that proper attention to such constraints can be critical. Mate selection solutions that break important constraints are generally difficult to fix "manually". Thus, the practitioner must be satisfied with the proposed solution.

The objective of this paper is to present a mate selection method that achieves such grouping constraints directly, without involving solutions that break the constraints, and to compare its performance with an existing approach that is based on penalising illegal solutions that arise during analysis. In order to present the new method, a full description of the underlying mate selection algorithm is provided since, to date, it has not been presented elsewhere, despite its relatively wide use.

## Method

Whenever the consequences of a particular mating set can be evaluated by simply summing the value of each mating carried out, we can use linear programming in a relatively simple manner to find the optimal mating set [5]. However, for most animal breeding problems, the value of a mating depends on which other matings are made. For example, the decision to mate a particular bull with a cow will be increasingly inhibited if the bull is used for an increasing number of other cows, as this will result in more inbreeding in the long term. Similarly, the value of mating a bull with cows in two different farms to increase genetic connection is decreased if many other such matings already give a good connection. Alternatively, if the aim of a given mating program is to generate bimodality of the genetic value for intramuscular fat, in order to target two different product markets, the mating value will decrease if most other matings have the same outcome. To handle such issues, we need a more flexible method that evaluates the impact of each complete mating set analysed.

The method to analyse mate selection used in this paper is based on an evolutionary algorithm, which loosely mimics a biological process evolving towards an optimal solution. The terms "generation", "genotype", "phenotype" and "fitness" will be used to help illustrate this method, and these should not be confused with similar terms used for the animal breeding application itself.

**Figure 1:**
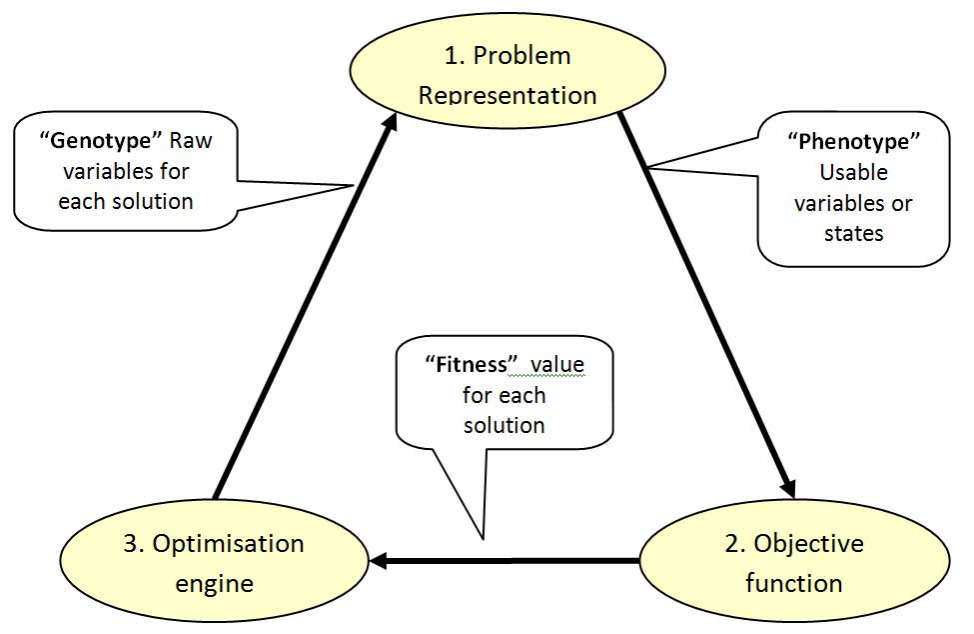
**The structure of an evolutionary algorithm **[7].

A mate selection analysis, as used in this paper, has three key components (Figure 1) that are used iteratively over "generations" to derive the optimal solution:

1. A problem representation component that uses a vector of numbers (analogous to a multilocus genotype) and translates these numbers to a representation of a solution (analogous to a phenotype), which in this case is a mating list.

2. An objective function component that evaluates each phenotype to calculate its fitness (analogous to selective advantage).

3. An optimisation component that uses the fitness value for each of the genotypes that it has produced to help select, mutate and recombine existing genotypes to provide new candidate genotypes.

A key advantage of this approach is that the optimisation engine is highly disjointed from the problem itself. It does not "know" or "understand" the problem, it simply delivers candidate solutions, in a raw form, and receives feedback on the value of each of these. This means that the problem itself can become increasingly complex, without the need to increase the complexity of the optimisation machinery. Importantly, the objective function can evaluate a whole mating set, including the types of interactions between matings described above.

Given this disjointed nature of the optimisation engine, the current paper does not include a detailed description of the optimisation engine that it uses to generate results. It is based on Differential Evolution (DE) [6], with adaptations described by [7].

### Strategies to apply constraints

Two strategies can be used to constrain the solutions (mating lists or "phenotypes") [7]:

• **Penalising: **Broken constraints are diagnosed within the objective function, and the resulting fitness value is penalised. A hard penalty is one that generally renders the solution uncompetitive for use by the optimisation engine to help make new candidate solutions. A soft penalty is less stringent, with penalties chosen such that solutions that break constraints are exploited earlier in the analysis, but become uncompetitive as an optimal solution is approached.

• **Fixing: **This strategy requires a more detailed treatment at the problem representation stage to ensure that no candidate solution (or "phenotype") breaks the constraint(s).

Penalising is generally easy to carry out. It only requires the diagnosis of constraint breakage for each solution, and ideally an extent of breakage. The latter is important whenever all initial solutions are illegal. In this case, rewarding the solutions that are less illegal with higher fitness values allows the method to move forward and eventually leads to legal solutions. This can, however, result in an analysis that effectively consists of two stages; if the range of possible fitness values for legal solutions is 0 to 1, then applying a 100 unit penalty for each broken constraint will lead to legality, but with little emphasis on the desired attributes of legal solutions. Once fitness values become positive, there will be progress towards a legal solution of high merit. However, during this second phase, a great deal of selection pressure can be taken up in maintaining legality, with typically most candidate solutions being of no value as they break one or more constraints, resulting in high computing times.

### Mate selection without grouping

The mate selection driver described in [8] can be used for simple scenarios that place no grouping constraints on the pattern of mating (Table 1). It gives a good example of translating "genotype" (the numbers underlined in Table 1) to "phenotype" (the tick marks, or mating list).

**Table 1:** A mate selection driver

			Female →	1	2	3	4...
Male ↓	*Nm*	Ranking criterion	Rank	**1**	**0**	**1**	**1**

1	**2**	**5.32**	2			✔	**✔**
		**2.16**	3				

2	**0**	**-**	-				

3...	**1**	**7.64**	1	**✔**			

The components to be optimised for mate selection are underlined. A tick denotes a mating to be made. *Nm *is the number of matings to be made for each individual. The Ranking criterion is used to find Rank, which defines the order of allocation of male matings to female matings [8].

Based on this mate selection driver: the underlined numbers in Table 1 drive the three matings noted, and these are the values to be optimised. *Nm *(second column for males, second row for females) is the number of matings for which each animal should be used, and this in turn drives selection, including the extent to which each animal is used. An animal is culled if this is set to zero. The ranking criterion is simply a real number assigned by the optimisation algorithm, one for each mating, and these numbers are ranked to give the column Rank. This is not a ranking on merit, but simply an order of presentation to drive the mate allocation part: The first ranked male mating is the single mating of male 3 and it is thus allocated to the first available female mating (the one nearest to the left) - the only mating of female 1. The second ranked male mating is the first mating of male 1 and it is thus allocated to the second available female mating (the one second nearest to the left) - the only mating of female 3. The third ranked male mating is the second mating of male 1 and it is thus allocated to the third available female mating - the only mating of female 4.

Notice that the mate allocation part of this simple algorithm breaks no constraints i.e. the row and column sums of matings match the numbers of matings (*Nm*) to be generated for each candidate. The optimisation engine operates with the underlined numbers "in ignorance" of this algorithm, except through eventual effects on fitness, just as the biological methods to select, mutate and recombine DNA operate "in ignorance" of the phenotypic outcome, except through eventual effects on fitness.

#### Constraints on number of matings per candidate

To invoke the mate selection driver of [8], we need to constrain *Nm *to declared limits for each candidate while achieving the targeted total number of matings (*Nt*). These constraints are presented here to help illustrate the application of the grouping algorithm later on. The one inevitable constraint is to have a non-negative *Nm *for each candidate, and this is easily achieved by using the "Fixing" strategy, constraining the raw solution variables to be non-negative. The other constraints that are usefully applied through the Fixing strategy are:

•Maxuse: The maximum value for *Nm*. For example, Maxuse = 1 mating for natural mating females, 30 matings for natural mating bulls, 1,000 matings for artificial insemination bulls, or the number of semen doses left for a deceased bull.

•Minuse: The minimum value for *Nm *given that the individual will be used at least partly. For example, if a bull is to be selected for natural mating, we might specify a minimum female group size of Minuse = 15 for that bull, as mating groups of less than this size may not acceptable to the breeder. In this case *Nm *= 0 is permitted, as are *Nm *> or =15.

•AbsMinuse: The absolute minimum value for *Nm*. This is generally zero, but may be set higher, for example when a breeder has a given number of semen doses available for a favoured bull, and insists that these should all be used.

The raw variables for *Nm *for each candidate are non-negative integers that are initially generated by the optimisation engine (Figure 1) but constrained to meet the above three limits, first with setting to 0 or Minuse for values between these, with a linearly greater probability of moving to the closer constraint, followed by setting to Maxuse or AbsMinuse for values that still violate one of these two constraints.

These constraints are maintained during an iterative process until ∑ *Nm *= *Nt*: while ∑ *Nm *is different from *Nt*, a candidate is chosen at random, and has one mating added (if ∑ *Nm *<*Nt*) or subtracted (if ∑ *Nm *>*Nt*), and this action is reversed when a constraint is violated. A slight modification is made to reduce the probability of allocating a mating to any male that has *Nm *= 0. This speeds convergence, as an optimal solution often has many males with *Nm *= 0.

### Mate selection with grouping: the GroupFix algorithm

The full mate selection algorithm, with grouping constraints, is referred to as GroupFix, as it uses a fixing strategy, rather than a penalising strategy, to ensure that group mating permission constraints are observed. Extra variables to be optimised are used to give relative weightings that help determine the target number of matings in each male by female group combination, and this works in conjunction with a mate selection driver to give solutions that are always legal.

This method should not be confused with the "Mate selection by groups" method [9], which does not involve grouping constraints. The motivation of the method in [9] is simply to speed computation, using cluster analysis to form multiple groups for each sex, then allocating *numbers *of matings at the level of these groups, followed by individual mate selection.

#### Weightings for target number of matings, W

Table 2 shows an example calculation of relative weightings (W), used to set the target number of matings for each group combination. For each female group, the aim is to reach a set of relative weightings, one weighting for each male group, that sum to one; these will be used to help set the target number of matings within each male group for the prevailing female group.

**Table 2:** Derivation of relative weightings (*W*) from raw weightings (*R*), the mating permission matrix and action types

Male Group	FG1	FG2	Female Group FG3	FG4	FG5
	Permission Matrix				
MG1	1	1	1	0	0
MG2	0	1	1	1	0
MG3	0	1	1	1	1
MG4	0	0	1	1	1

	Action type				
MG1	1	Opt	Opt	.	.
MG2	.	Opt	Opt	Opt	.
MG3	.	Opt	Opt	Opt	Opt
MG4	.	.	Calc	Calc	Calc

	Raw weights (*R*)				
MG1	1	0	0.3	.	.
MG2	.	0.2	0.6	0.2	.
MG3	.	0.1	0.6	0.3	0.8
MG4	.	.	.	.	.

	Relative weights (*W*)				
MG1	1	0	0.15	.	.
MG2	.	0.667	0.3	0.16	.
MG3	.	0.333	0.3	0.24	0.8
MG4	.	.	0.25	0.6	0.2

A'1' in the permission matrix denotes that matings can be made between the groups concerned; raw weights *R *are set by the optimization algorithm; relative weights *W *are used to help set the number of target matings per group combination; action types indicates whether the weights for that mating combination are set (1), optimized by the optimization algorithm (Opt) or calculated from weights for the other mating combinations (Calc).

A permission matrix shows which group combinations are permitted for mate allocations, with 1 for permission and 0 for no permission. The action type for each male × female group combination depends on the permission matrix. For a given female group:

• There is no action (denoted by a period) wherever permission = 0.

• If only one male group is permitted the action type is 1 for that group and the final relative weighting is 1.

• Otherwise, the action type is "Opt", denoting that an optimal raw weighting value (*R*) has to be found by the optimisation engine, for all permitted male groups except the last male group.

• If the last male group is permitted, and one or more other male groups are also permitted, its action type is "Calc", meaning that its relative weighting is to be calculated as shown below.

This means that the number of raw weightings (*R*) to be optimised to manage grouping is between zero, when only one male group is permitted for each female group, and (number of female groups) × (number of male groups -1), or *N_FG_*(*N_MG _*- 1).

Table 2 shows an example set of values from the optimisation engine, which are used as raw weightings (*R*). Each of these has been constrained to between 0 and 1 by truncation. Relative weightings (*W*) for the i, j^th ^male, female group are computed from the raw weightings as:

for *i *<*N_MG _*and when the last male group is not permitted: W_i, j _= R_i, j_/∑R_., j_;

for *i *<*N_MG _*and when the last male group is permitted: ${\text{W}}_{\text{i,j}}={\text{R}}_{\text{i,j}}/\left(1+\frac{({\text{k}}_{\text{j}}-2)\sum {\text{R}}_{\text{.,j}}}{K_{\text{j}}-1}\right)\;\;;$

for *i *= *N_MG _*: ${\text{W}}_{\text{i,j}}=\frac{\left(1+\frac{({\text{k}}_{\text{j}}-2)\sum {\text{R}}_{\text{.,j}}}{K_{j}-1}\right)\;-\;\sum {\text{R}}_{\text{.,j}}}{1+\frac{({\text{k}}_{\text{j}}-2)\sum {\text{R}}_{\text{.,j}}}{{\text{k}}_{\text{j}}-1}},$

where *k_j _*is the number of positive raw weightings, plus 1 if the last male group is permitted. The last male group is treated differently because it has no raw weightings, and its relative weighting is contingent on the raw weightings for the other male groups.

With reference to Table 2, this gives the following sensible outcomes:

• All columns of *W *sum to one.

• When the mean value of all R > 0 is 0.5, *W *for the last male group, if permitted, is the average of *W*.

• When the mean value of all R > 0 is < 0.5, *W *for the last male group is above the average of *W*, and vice versa.

• When all R = 0, W for the last male group = 1.

• When one or more R = 1 and the rest = 0, *W *for the last male group, if permitted, = 0.

These results give an efficient coverage of relative weightings to be used for target number of matings per group combination, with a minimal number of raw weightings to be optimised.

The next set of steps will define the target number of matings to be carried out within each male by female group combination for the current solution. These are driven by *Nm *values for individual candidates, as in Table 1, plus a raw weighting (*R*) for each group × group combination that is marked "Opt" in Table 2. This will be followed by individual mate allocations using the ranking criterion values, one per male candidate, as in Table 1, to satisfy these target numbers for the current solution. Notice that *Nm *values, ranking criterion values and *R *values are supplied for each solution by the optimisation engine (Figure 1).

#### Target number of matings per group × group combination

##### Constraints on the number of matings per female group

For each female group, the target number of matings for the whole group is the product of the number of candidates and the selection proportion declared by the user for that group. [It is also possible to optimize the selection proportions by adding them to the list of parameters to be optimised, effectively giving an optimised multistage selection scheme]. Constraining the total number of matings for each female group to match this target follows the iterative process of adding/subtracting matings from individual candidates, as described above for the no-grouping case.

##### Initial target number of matings per group × group combination

The target number of matings for each group combination is then initiated. For each female group *j*, the target number of matings with each male group *i *is set using the weightings *W *described above, giving *Nmg *as the number of matings for each group × group combination:

$$Nmg_{i,j}=W_{i,j}Nt_{j}$$

with additional steps to ensure integer outcomes, using *W *to set the probabilities of each group being perturbed to give equality.

##### Constraints on the number of matings per male group

The *Nmg *values can break constraints on male use, for example where ∑ Nm_i,. _exceeds the sum of maximum use of the males from group *i*. This is handled by iteratively reallocating target matings from the male group that breaks a constraint to another randomly chosen male group that can accept the change required from it, with this reallocation taking place within a female group that can accept the change at both the source and destination male groups.

Given *Nmg *values that do not break overall male use constraints, the total number of matings for each male group is then constrained to match this target following the iterative process of adding/subtracting matings from individual candidates, as described above for the no-grouping case and for females in the grouping case.

At this stage, we have the number of matings to be allocated to each candidate of each sex, together with a target number of matings for each group combination. The next step is to make the individual mate allocations.

#### Individual mate allocations

The optimisation engine provides a ranking criterion for each male mating, as in Table 1. Typically each male has zero or multiple matings to make, and there is a ranking for each mating, rather than for each male, such that matings for a given male are generally dispersed throughout the ranked list.

For the current solution to be evaluated for the objective function, male matings are accessed sequentially according to their position in this ranked list. Each male mating is allocated to the next available female mating (from left to right on row 2 in Table 1) that is both unallocated and legal according to group permissions. For this purpose, female matings can be listed in an arbitrary order that is fixed for the duration of the analysis. However, sorting the female list on attributes of importance in the objective function tends to speed up convergence, as this provides a smoother response surface for the optimisation engine to climb. Moreover, optimising the order of accessing female matings increases the flexibility of covering the response surface, making valleys to be crossed less deep. When this process is completed, the number of individual mate allocations within each group combination will match the target set for each group combination.

This method works for oocyte harvesting with *in vitro *fertilisation (IVF), or indeed in fish species where IVF is easily managed, since the multiple matings of a single female can each be covered by a different male. However, a slightly different treatment is required for cases involving *in vivo *fertilisation following superovulation, as in classical multiple ovulation and embryo transfer (MOET) practices. The male assigned to the first mating to be allocated to a MOET female has to be used for all her remaining matings.

### Testing method

The GroupFix algorithm was tested by comparing its speed and pattern of convergence with a penalising strategy. Various penalties were applied in the latter for solutions that break one or more grouping constraints.

An example dataset was generated using PopSim, available at http://www-personal.une.edu.au/~bkinghor/genup.htm. Three separate breeding farms each mated 25 males to 100 females each year with: the first progeny born when parents were 3 years old; culling for age after 5 (8) mating cycles for males (females); selection on an economic index using BLUP EBV; random adult annual survival of 95%; and a 80% calving rate for females. These breeding programs were set up with a complete age structure and then run for ten mating cycles.

The problem tackled here was to set up the next mating round, across farms. All live males and females of appropriate age were considered as candidates for selection. There were 443 male candidates and 596 female candidates with a requirement to make 341 matings across farms and groups, of which 287 matings were in the active mating group combinations that do not involve juveniles or embryos (see Table 3).

**Table 3:** Group mating permission matrix for the test dataset

		Female group				
		Farm 1	Farm 2	Farm 3	Juvenile	Embryo
Male group	Farm 1	1	0	0	1	0
	Farm 2	0	1	0	1	0
	Farm 3	0	0	1	1	0
	Juvenile	1	1	1	1	1
	Embryo	0	0	0	1	1
	AI	1	1	1	1	0

Farm denotes the farm of birth, embryos are animals already conceived in the current year, juveniles are animals conceived in the previous year; bulls that can be used for artificial insemination (AI) are defined as having already been used for one or more mating cycles; a '1' denotes that matings can be made between the groups concerned; in this case, no migration between farms is permitted for natural mating purposes; matings involving embryos or juveniles are virtual matings and not part of the active mating set.

Table 3 shows the group mating permission matrix that was used. This matrix is formed by the practitioner and this can involve some subjectivity, for example in the rules that define which bulls are used for artificial insemination. This example involves non-active 'virtual' matings, which are produced by the analysis but not intended to be implemented in reality.

Virtual matings involving existing juveniles and predicted embryos (as predicted from the previous mating round) can be useful to include in the analysis, for example to help inhibit the high use of a bull in the current mating round which has already contributed greatly to the next generation, as evidenced by the number of juvenile and embryo progeny.

The penalising strategy was invoked by reducing the fitness of a solution by a weighting factor times the number of matings that take place within group combinations that contain a zero in the group mating permission matrix. Weightings used were 100, which in this case effectively make the rest of the objective function irrelevant for illegal solutions, and lower weightings were used in different treatments to give softer constraints, *viz*. 0.1, 0.01, 0.005 and 0.001.

#### Objective function

The objective function used for the test example was a function of the mean EBV index of the predicted progeny, the coancestry among the parents used in the mating set, weighted by their use, and the mean inbreeding of the predicted progeny. A general description is given here, with details in Additional file 1, appendix.

**Figure 2:**
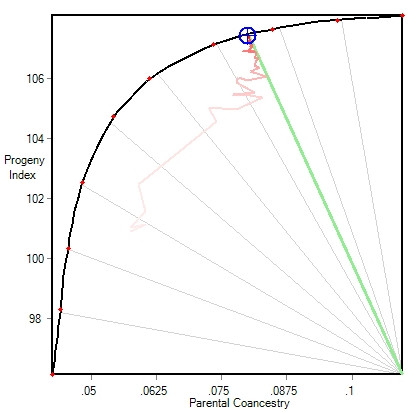
**An example frontier response surface involving Progeny Index and Parental Coancestry**. See text for details; from the MateSel tool in Pedigree Viewer, available at http://www-personal.une.edu.au/~bkinghor/pedigree.htm.

The relative emphasis on the mean index versus coancestry was set in the light of their response surface (Figure 2). The curved frontier in this figure shows the range of possible outcomes of optimal contributions (number of matings allocated to each candidate), with each point reflecting a different relative weighting on mean progeny index versus parental coancestry [see [10]]. However in this case, the frontier accommodates the grouping constraints in Table 3, using the GroupFix algorithm for all treatments, so that the same conditions prevail for each treatment during its main run.

The software used to run the current tests can manage the balance between mean index and parental coancestry in several ways. Here we used a target of 25 degrees, where 0 degrees corresponds to the maximum progeny index response and 90 degrees to minimum parental coancestry (see Figure 2). An optimal solution has been reached at the point on the frontier that corresponds to 25 degrees (Figure 2), with the trailing path showing the progress of the DE algorithm towards this point.

When other component criteria are included in the objective function, such as progeny inbreeding, the frontier point is generally not reached. However, the software used manages the outcome such that the optimal solution will lie close to the target 25 degree line in Figure 2. In this study, progeny inbreeding was given a moderate negative weighting of -1, or a zero weighting, as described below.

## Results

**Figure 3:**
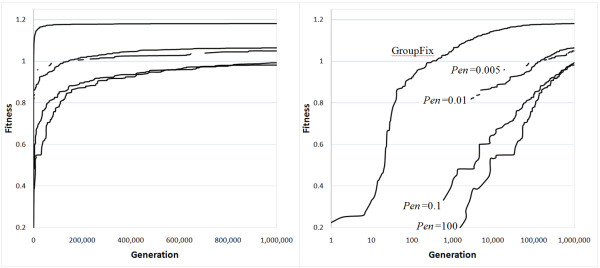
**Fitness of the best solution by generation of the DE algorithm for different strategies**. This figure censors results for those strategies and generations in which the best solution breaks a constraint, and this is seen as gaps in the plot for each strategy; the right-hand graph gives generation on a logarithmic scale to help differentiate the strategies; the strategies are GroupFix and the four penalising strategies denoted by their penalty weighting, Pen, as labelled on the right-hand graph. Strategies Pen = 0.01 and Pen = 0.005 cross over at about generation 150,000.

Figure 3 shows fitness of the best solution by generation of the DE algorithm for each strategy, with a weighting of -1 for progeny inbreeding. The best solution in the first generation of the evolutionary algorithm for the Groupfix method gave values of 7.30, 0.0054 and 0.0076 for the mean progeny index, mean progeny inbreeding and mean parental coancestry, with the latter figure being low due to essential panmixia. In generation one million of the Groupfix algorithm, these figures were 10.53, 0.0021 and 0.0485. The GroupFix strategy converged essentially after about 100,000 generations, when it had reached 99.5% of the fitness from generation one million compared to the fitness from generation one (itself the best of 50 randomly generated legal solutions). This stage was reached in 3559 seconds on a 2.4 GHz laptop computer. At this stage, the best penalising strategy was 78.5% converged, which was reached by the GroupFix strategy by generation 216. None of the penalising strategies converged even close to the optimal solution after one million generations of the DE algorithm, with regular small improvements still being made up to that stage. Of course the optimal solution and maximal fitness are the same for all strategies, illustrating that the penalising strategies performed very badly indeed. In fact, the best of these strategies at one million generations (23,327 CPU seconds) had a lower fitness than the GroupFix strategy had reached by generation 1057 (29 CPU seconds).

A lower penalty weighting allows some evolution towards a useful solution simultaneously with the process of developing legal solutions. This can be seen by the higher fitness for lower weightings in earlier generations in Figure 3. In later generations, fitness is also higher for lower weightings, except for the lowest weighting strategy (weight = 0.005). This is likely because the direction of evolution while illegal solutions prevail is not fully appropriate to that under full legality, and overall progress in fitness becomes impaired for this strategy because of the long periods in which legality is absent.

With a very small weighting of 0.001 on illegal solutions, no legal solution features as the most-fit solution in the one million generations that these analyses were run for. It is essentially not possible to predict the best weighting to use in a penalising strategy, such that some testing would be required for each problem.

For this example, the negative weight on progeny inbreeding is the only component in the objective function that impacts the mate allocation part of the mate selection algorithm. Setting this weighting to zero renders the pattern of mate allocation inconsequential, given that group legality is maintained. Under these circumstances, convergence is generally quicker; in this case, the GroupFix strategy had reached 99.5% of the optimal solution after 46,659 generations. At this stage, the best penalising strategy was 70.7% from the optimal solution, which was reached by the GroupFix strategy by generation 81. The best penalising strategy at one million generations (24,071 CPU seconds) had a lower fitness than the GroupFix strategy had reached by generation 2325 (78 CPU seconds).

## Discussion

Various mate selection algorithms have been described in the literature, with differing levels of functionality. Analysis based on linear programming [5] works when the value of a mating is independent of which other matings are done. However, this does not cover issues such as parental coancestry or connection between herds, where the whole portfolio of matings must be evaluated. Simulated annealing [11] and evolutionary algorithms [8,7,12] have been used to address this shortcoming, as well as a two-step approach of selection followed by mate allocation [13]. However, none of these methods allow inclusion of grouping constraints, as described in this paper.

The GroupFix method generates candidate mate selection solutions that do not break declared grouping constraints and gives much improved flexibility and robustness in mate selection operations compared to other methods.

As noted by one referee, no general proof is offered that the GroupFix algorithm accesses the full legal solution space. However, a test was carried out whereby a legal solution was produced independently from the GroupFix algorithm. This was treated as if it were an optimal solution that was to be found by the GroupFix algorithm, by using an objective function that compared the current mate selection set to this "optimum" mate selection set. The GroupFix algorithm was successful in finding this solution.

The GroupFix algorithm has been used extensively since 2007 in several operational breeding programs, with the biggest runs involving several thousand candidates for selection. It produces a dramatic increase in speed of mate selection analyses for scenarios that involve at least a moderate degree of grouping constraint. In this study, the alternative penalising strategies were several hundred times slower, and in fact none of these approached reasonable convergence for the scenarios tested.

The GroupFix method is important for application of mate selection methods that integrate decision making across issues in progressive breeding programs. It gives a general framework for setting and managing the types of grouping constraints that animal breeders would like to impose. It also enables accommodation of overlapping generations by including groups that constitute the complete age structure and life cycle of animals, including for example embryos and pregnant females, along with candidates for the active mating group. This is an alternative to other approaches for handling overlapping generations [14,15].

Another prospect of the method is running mate selection analyses simultaneously across multiple herds. This gives opportunity to manage issues such as quarantine barriers and transport costs, for example by reducing the fitness of a solution by a weighting factor times the total transport distance that the solution dictates for live bulls. Policies on managing issues such as direction of genetic change, genetic diversity, genetic variation for specified traits, and gene marker profiles can be set or influenced at a regional or breed level. For example, the association for an endangered breed might set a policy recommendation to set the target degrees in Figure 2 at 35 degrees, to give more emphasis to genetic diversity.

For complex runs involving many issues, it is useful to adjust weightings and other controlling factors in a dynamic fashion. An example would be to change the target from 25 degrees to 35 degrees in Figure 2 during the analysis, and observe the impact on all component outcomes. This gives opportunity to explore the overall response surface and discover what outcomes are possible, before settling on a mating list to be adopted.

The analyses carried out in this paper used the author's program MateSel, with some additions to permit test runs based on penalising illegal solutions. MateSel executable code is freely available as part of the Pedigree Viewer program at http://www-personal.une.edu.au/~bkinghor/pedigree.htm

## Conclusions

The GroupFix method presented enables the use of mate selection for the implementation of progressive breeding programs in a wide range of scenarios, including programs across breeding units, with attention paid to the genetic and practical issues involved.

## Competing interests

The author declares that he has no competing interests.

## Supplementary Material

Additional file 1 **Appendix: Objective function details**. Objective function details referred to in the text. Click here for file
