# Supplementary material for: An algorithm for efficient constrained mate selection
Source: Genet Sel Evol. 2011 Jan 20;43(1):4. doi: 10.1186/1297-9686-43-4 (PMC3037843; doi:10.1186/1297-9686-43-4)
Supplement: Additional file 1 — Appendix: Objective function details. Objective function details referred to in the text. [file 1297-9686-43-4-S1.DOCX]

# Appendix: Objective function details

Based on the general concepts described in the text for weighting the mean progeny index, parental coancestry, and progeny inbreeding, the specific objective function (*OF*) to be maximised in the example was as follows:

If $aTan\left[ \frac{\left( x_{0}^{'}Ax_{0}-x^{'}Ax \right)/\left( x_{0}^{'}Ax_{0}-x_{90}^{'}Ax_{90} \right)}{\left( x^{'}G-x_{90}^{'}G \right)/\left( x_{0}^{'}G-x_{90}^{'}G \right)} \right]<TD$
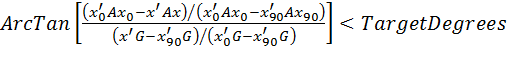


then $OF= \frac{\frac{x^{'}G}{2M}- \frac{x_{90}^{'}G}{2M}}{Cos\left( \mathrm{TD} \right)*\left( \frac{x_{0}^{'}G}{2M}- \frac{x_{90}^{'}G}{2M} \right)} -1.F$
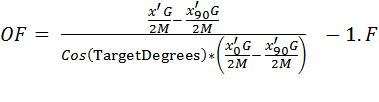


where $F= \frac{\sum_{i=1}^{M} 0.5a_{{Male}_{i},{Female}_{i}}}{M}$
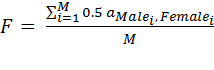


or else $OF=-{10}^{20}- \frac{x^{'}Ax}{8M^{2}}$
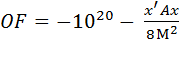


where *x* is the vector of contributions from male and female candidates, expressed as number of matings allocated to each, such that *x* sums to 2*M*, where *M* is the total number of matings to be made; *x_0_* is the vector of optimal contributions that maximize the progeny index and *x_90_* is the vector of optimal contributions that minimize parental coancestry (*x_0_* and *x_90_* having been determined by this stage, *0* and *90* relate to degrees in Figure 2); *a* is an element from the numerator relationship matrix *A*; *G* is the vector of candidate index or EBV values; *F* is the mean inbreeding coefficient in progeny that would result from the current mate selection solution, as defined by the parents (${Male}_{i},{Female}_{i})$ of the *i^th^* mating; TD = TargetDegrees is the degree line, set to 25 degrees in Figure 2, below which value a solution is taken to be illegal. The latter is effected with an *OF* of -10^20^ but with an additional penalty on high coancestry to help approach legality in the case that all solutions are illegal. Notice that for legal solutions, the mean predicted progeny merit
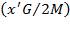
$\left( {x^{'}G}/{2M} \right)$ is expressed as a deviation from the minimum merit previously found with full emphasis on reduced coancestry
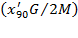
$\left( {x_{90}^{'}G}/{2M} \right)$, and then scaled by a denominator that gives an expected range from 0 to 1 after scaling, assuming a circular shape for the frontier in figure 2. This is done in order to give a more consistent impact of given weighting values on other components in the objective function, such as the chosen weighting of -1 on progeny inbreeding in *OF* above. Adoption of such a strategy in developing components of the objective function helps to give more consistent outcomes across different runs, for example for different farms, or for the same population at different mating cycles. This is an important step towards ‘automated’ analyses that require little or no human intervention.
